# A Technical Framework for Predicting Regulatory Deficiencies in Pharmaceutical Chemistry, Manufacturing, and Controls (CMC) Submissions Using Machine Learning

**DOI:** 10.7759/cureus.110534

**Published:** 2026-06-09

**Authors:** Piyush Modi

**Affiliations:** 1 Regulatory Affairs CMC (Chemistry, Manufacturing, and Controls), Amneal Pharmaceuticals of NY LLC, New York, USA

**Keywords:** cmc analysis, drug development, healthcare technology, machine learning, natural language processing, pharmaceutical quality, regulatory deficiencies, regulatory intelligence, regulatory submissions, risk prediction

## Abstract

Regulatory review of Chemistry, Manufacturing, and Controls (CMC) information can reveal issues with stability, impurities, specifications, manufacturing controls, analytical methods, or process validation. These problems may lead to extra review cycles and approval delays. This report introduces a conceptual machine-learning framework to support the identification of potential CMC deficiency risks prior to submission. The framework brings together document ingestion, natural language processing, a regulatory knowledge base, predictive classification, risk categorization, and recommendation generation. It outlines possible methods and provides an example use case to show how the system could work. No regulatory submission data were analyzed, no model was trained, and no predictive results were measured; therefore, all examples are hypothetical and not validated. Future work needs to focus on developing models with appropriate data, comparing results with expert reviewers, validating externally and prospectively, and handling confidentiality, cybersecurity, explainability, and regulatory acceptance.

## Introduction

Chemistry, Manufacturing, and Controls (CMC) information explains how a pharmaceutical product and its components are developed, manufactured, tested, controlled, packaged, and maintained in a stable condition over time. Regulatory deficiencies can result from missing stability data, poorly justified specifications, insufficient impurity qualification, unclear manufacturing details, weak analytical method validation, or incomplete process validation information [1,2]. Regulatory review deficiencies can represent a significant proportion of review observations and are associated with increased development timelines.

Today, CMC reviews rely primarily on experts who manually assess large volumes of both structured and unstructured information. Artificial intelligence (AI) and machine learning (ML), especially natural language processing (NLP), can help extract information, classify documents, and identify patterns [3-6]. However, most published work has focused on healthcare prediction or general text analysis, not on a CMC-specific system that connects document features to past deficiency patterns and suggested fixes.

This report proposes a conceptual technical framework to help identify CMC deficiency risks using machine learning prior to submission. The goal is to outline a testable system design and a path for future validation, not to present a finished or validated product.

## Technical report

This work did not analyze any real regulatory submissions, nor was any machine learning model trained or tested. The following framework outlines planned components, potential technical methods, and outputs that will require future development and validation. Table 1 shows how the framework integrates data processing, feature extraction, historical knowledge, and predictive analysis to assess risk and provide recommendations.

**Table 1:** Components of the proposed conceptual framework CMC: Chemistry, Manufacturing, and Controls; NLP: Natural Language Processing; eCTD: electronic Common Technical Document

Component	Function
Data ingestion module	Converts PDF, Word, eCTD, and XML content into machine-readable text and metadata.
NLP extraction engine	Identifies CMC concepts, including stability duration, impurity limits, specifications, process parameters, analytical validation, and supporting evidence.
Regulatory knowledge base	Stores structured historical regulatory data including deficiency records and inspection findings
Machine learning engine	Predicts probability of regulatory deficiencies using trained classification and ensemble models
Risk scoring module	Combines predicted probability, potential severity, and evidence completeness into prioritized risk categories.
Recommendation engine	Maps identified risks to traceable, expert-reviewed corrective actions.

The system works with regulatory submission files in formats like PDF, Word, eCTD, and XML. These documents often have unstructured information about stability data, impurity profiles, manufacturing details, and control protocols. The data ingestion module changes these files into structured formats for analysis. The engine uses NLP techniques to find and extract important regulatory details, such as stability indicators, impurity limits, process variables, and control procedures. This information is then organized into structured datasets for further analysis.

A regulatory knowledge base keeps historical regulatory data such as deficiency records, inspection results, and past submission outcomes. This organized data enables the system to compare new submissions against past patterns and support predictive analysis.

**Figure 1:**
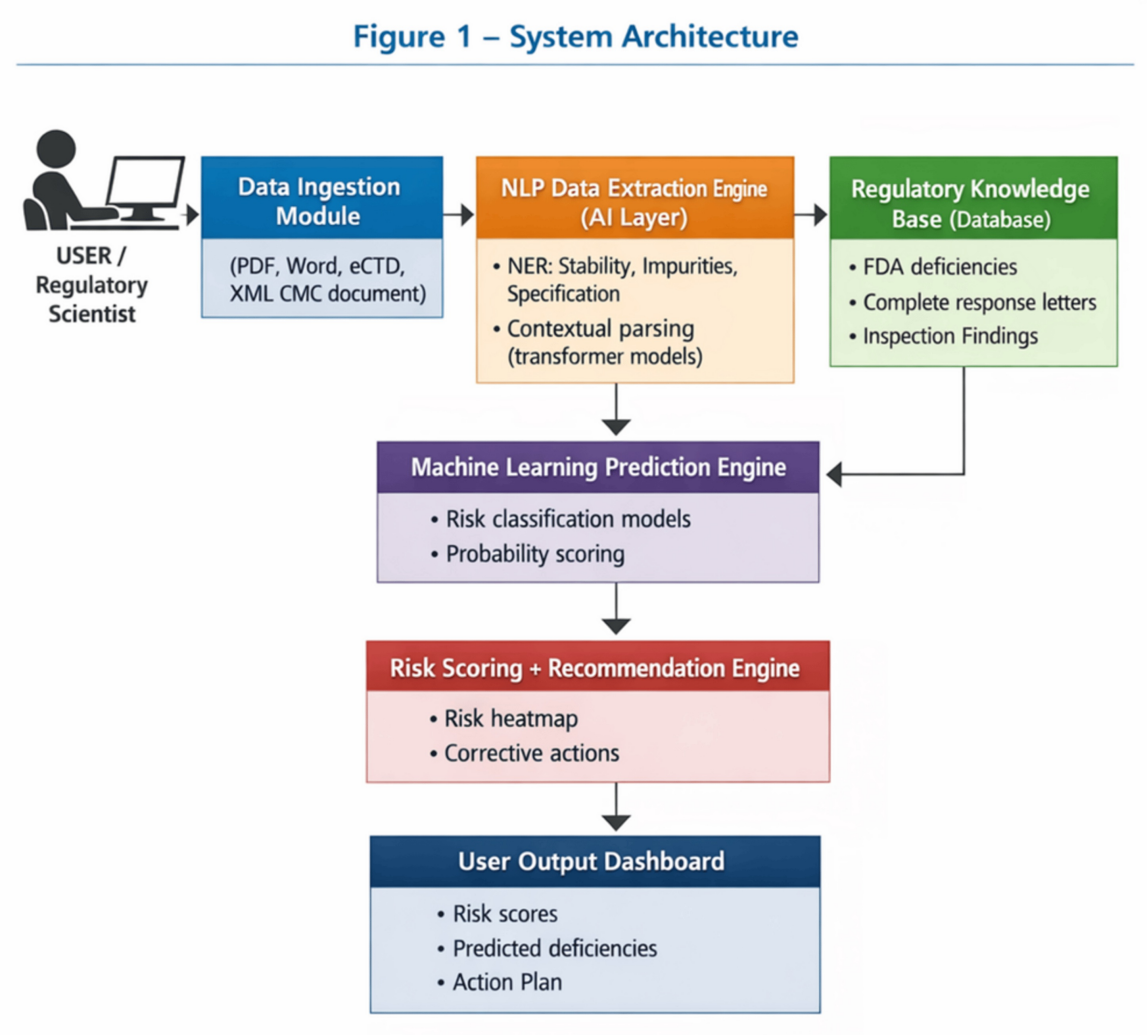
Conceptual architecture of the proposed machine-learning-based regulatory deficiency risk framework AI: Artificial Intelligence; CMC: Chemistry, Manufacturing, and Controls; eCTD: electronic Common Technical Document; FDA: Food and Drug Administration; NER: Named Entity Recognition; NLP: Natural Language Processing; XML: Extensible Markup Language. Figure adapted from the author’s provisional patent application (Computer-implemented system for predicting regulatory deficiencies in pharmaceutical CMC submissions using machine learning. Unpublished U.S. Provisional Patent Application 64/071,028; filed May 21, 2026), and created using Microsoft PowerPoint (Microsoft Corporation, Redmond, WA, USA).

The machine learning prediction engine examines the structured data from feature extraction and compares it against the regulatory knowledge base. The models are intended to produce probability-based forecasts of potential regulatory deficiencies across different sections of the submission. The risk-scoring module calculates both overall and section-specific risk scores based on predicted deficiencies. These scores highlight high-risk areas and help set priorities. The recommendation engine provides practical advice to improve submission quality. Based on predicted risks, the system suggests actions such as adding missing data, clarifying specifications, or providing more documentation. Figure 1 shows the architecture of the machine learning framework, including data ingestion, NLP extraction, prediction, risk scoring, and recommendation components. Please note that the figure illustrates the intended workflow only and does not represent an implemented or validated system.

The overall system architecture and functionality described in this section constitute the author’s proposed framework for predictive regulatory assessment and align with the system described in the associated patent application. The patent filing does not constitute evidence of implementation, predictive validity, or regulatory acceptance. 

Illustrative application of the proposed framework

Since the framework has not yet been built, this section provides only hypothetical examples. These show the intended output format and should not be seen as real model predictions or scientific results. Table 2 illustrates hypothetical observations, corresponding risk classifications, and potential recommendations, demonstrating the intended application of the proposed framework.

**Table 2:** Hypothetical use cases illustrating intended outputs; no trained model generated these classifications

Hypothetical observation	Illustrative risk	Potential recommendation
Long-term stability data are absent	High	Provide available long-term data and clarify the post-submission stability commitment.
An impurity limit is stated, but its scientific or toxicological justification is unclear.	Moderate	Provide justification based on qualification data, process capability, compendial expectations, or relevant regulatory guidance.
Process-validation information contains the defined protocol, acceptance criteria, and supporting evidence.	Low	Confirm consistency across modules; no immediate corrective action may be required.

To demonstrate the conceptual application of the proposed framework, illustrative scenarios are presented. These examples illustrate how the system is designed to evaluate regulatory submission content and identify potential areas of concern using extracted features and historical patterns.

As illustrated in Table 2, the framework may classify sections with missing long-term stability data as higher risk and suggest providing additional supporting data or clarifying post-submission commitments. Similarly, impurity specifications lacking clear scientific justification may be identified as moderate risk areas where further clarification is recommended. In contrast, sections containing comprehensive process validation information may be considered lower risk, with minimal additional action required beyond consistency checks.

Based on these illustrative assessments, the framework is intended to generate overall and section-specific risk insights along with corresponding recommendations. These examples are hypothetical and are provided to demonstrate the framework's intended functionality; no empirical validation has been performed.

Future development and validation

To implement this, a governed dataset of de-identified and properly authorized regulatory records is needed. The dataset should include details like source, time period, submission type, deficiency category, class balance, labeling rules, and data quality checks. Independent regulatory experts should label the cases, and any disagreements should be settled through adjudication, with the levels of agreement reported.

A future study should use separate training, validation, and test sets, and, if possible, validate externally with data from a different time or organization. Performance should be measured by sensitivity, specificity, precision, recall, F1 score, area under the receiver operating characteristic (ROC) curve, calibration, and confidence intervals. When comparing results with expert reviewers, check agreement, review time, and the impact of false positives and false negatives. Prospective testing should happen before the system is used in practice.

## Discussion

The proposed framework is intended to demonstrate how machine learning and natural language processing could help identify risks of regulatory deficiencies in pharmaceutical CMC submissions before they occur. Earlier studies have shown that machine learning can help with prediction and decision-making in healthcare and related areas [3,4]. Advances in natural language processing, especially transformer-based models, have made it easier to extract structured information from unstructured text [5,6]. This framework brings together NLP-based feature extraction, a regulatory knowledge base, machine learning risk estimation, and a recommendation module. In the future, these parts could help spot incomplete or inconsistent CMC information and suggest review prompts to help regulatory professionals improve submissions. However, these features have only been proposed and have not yet been tested in real regulatory submissions.

Parts of the framework are also described in the author’s provisional patent application (Computer-implemented system for predicting regulatory deficiencies in pharmaceutical CMC submissions using machine learning. Unpublished U.S. provisional patent application 64/071,028; filed May 21, 2026). This is shared for transparency and does not prove the system works or is accepted by regulators. The approach matches established pharmaceutical quality and risk-management principles. International Council for Harmonization (ICH) Q10 highlights the need for strong quality systems throughout a product’s life, and ICH Q9 supports structured, science-based risk management [7,8]. By organizing CMC information and highlighting areas for further review, the framework could support these principles. Unlike traditional review processes that rely on manual document checks, this approach aims to use analytics to identify risks and help prioritize reviews. Predictive analytics has shown value in other areas of healthcare [9], but its use in identifying CMC deficiencies still requires further testing.

Limitations

The framework has several key limitations. It is still conceptual, with no regulatory data analyzed, no model trained, and no performance measures like accuracy, sensitivity, specificity, precision, recall, F1 score, or area under the ROC curve reported. Its future reliability will depend on the amount, quality, and consistency of historical regulatory data. Incomplete, biased, or organization-specific data could limit its effectiveness across different products, regulatory paths, and regions. False positives could lead to unnecessary reviews, while false negatives could give a false sense of security and cause important issues to be missed. So, the framework should be seen as a decision-support tool, not a replacement for expert judgment. Confidentiality and cybersecurity are also important since CMC submissions include sensitive manufacturing and product information. Future use would need safe data handling, controlled access, audit trails, explainable results, and ongoing human monitoring.

Future research should focus on building a well-managed, de-identified regulatory dataset, training and adjusting candidate models, and comparing their results with expert reviewers. Validation should include both internal and external tests, checking false-positive and false-negative rates, model calibration, and comparing with standard review methods. Integrating with electronic Common Technical Document systems and current regulatory workflows could make the system more useful, but this should only happen after proper technical validation, data security checks, and clear rules for model governance and human monitoring. This article proposes a testable conceptual framework, not proof that the system improves submission quality, predicts outcomes, or shortens review times.

## Conclusions

This technical report introduces a conceptual machine learning framework to help identify possible regulatory deficiency risks in pharmaceutical CMC submissions. The system combines NLP-based feature extraction, a regulatory knowledge base, predictive modeling, risk categorization, and recommendation generation. There has been no real-world validation yet, so no claims can be made about accuracy, improved submission quality, or reduced regulatory delays. Future work should develop a representative dataset, compare results with expert reviewers, validate externally and prospectively, and assess confidentiality, cybersecurity, explainability, governance, and regulatory acceptance prior to practical use.
